# Fungal Infections in Disorders of Inborn Errors of Immunity

**DOI:** 10.1007/s12016-026-09182-2

**Published:** 2026-07-16

**Authors:** Manpreet Dhaliwal, Vaishali Thakur, Adilia Warris, Gordon D. Brown, Rakesh Kumar Pilania, Ivy M. Dambuza

**Affiliations:** 1 Pediatric Allergy Immunology Unit, Department of Pediatrics, Advanced Pediatrics Centre, Postgraduate Institute of Medical Education and Research, Chandigarh, India; 2 MRC Centre for Medical Mycology, Faculty of Health and Life Sciences, Department of Biosciences, University of Exeter, Exeter, EX4 4QD UK; 3 Wellcome-Wolfson Institute of Experimental Medicine, Queen’s University Belfast, Belfast, BT9 7BL UK

**Keywords:** Inborn errors of immunity, Primary immunodeficiency diseases, Invasive fungal infections, Opportunistic mycoses, Chronic mucocutaneous candidiasis, Aspergillosis, Endemic mycoses, Antifungal immunity, Genetic susceptibility

## Abstract

Emerging infectious threats continually challenge the adaptability of the human immune system. Inborn errors of immunity (IEIs) provide a unique framework to dissect host-pathogen interactions, revealing how single-gene defects uncover critical pathways in antifungal defence. This review offers an overview of fungal immunity through the lens of IEIs, and integrates recent advances in innate fungal recognition, neutrophil and mononuclear phagocyte effector mechanisms, CARD9/NF-kB signalling, IL-17/IL-23 and IL-12/IFN-gamma cytokine axes, and transcription-factor defects that shape susceptibility to *Candida*, *Aspergillus*, *Pneumocystis*, *Cryptococcus*, dimorphic fungi and other moulds. We also discuss acquired phenocopies mediated by anti-cytokine autoantibodies, translate these mechanisms into clinical red flags and diagnostic algorithms, and discuss potential therapeutic strategies. Finally, we highlight how genetically defined IEIs can be used to study fungal-bacterial, fungal-mycobacterial and fungal-viral co-infections, revealing the hierarchy and plasticity of host defence in health and disease.

## Background

**Fig. 1:**
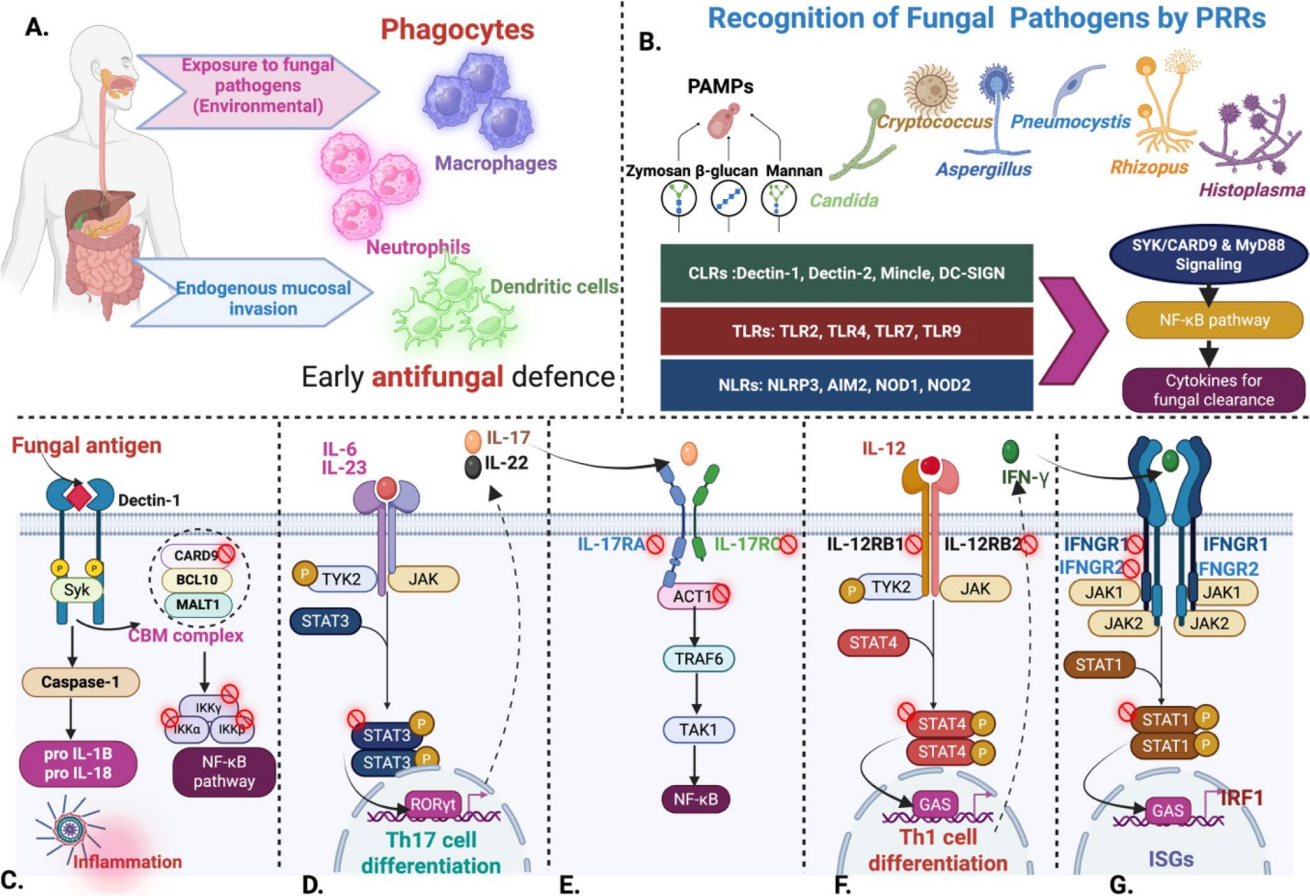
Genetic defects in inborn errors of Immunity predisposing to fungal infections. This figure illustrates key genetic defects and their downstream pathways in inborn errors of immunity (IEI) that increase susceptibility to fungal infections. Genetic defects in IEI compromise both innate and adaptive antifungal immunity, disrupting defences from pathogen recognition to Th1/Th17 effector responses, thereby predisposing individuals to fungal infections. Fungal pathogens are recognized through their PAMPs (zymosan, mannan, and β-glucan) by PRRs (TLRs, CLRs, and NLRs), leading to innate immune activation. The CBM complex acts as a central adaptor downstream of CLRs, linking fungal pathogen sensing to activation of the IKK complex (IKKα, IKKβ, IKKγ), which in turn activates NF-κB and drives transcription of pro-inflammatory and antifungal genes. IL-23-driven JAK-TYK2-STAT3 signalling promotes Th17 differentiation via RORγt, with IL-17RA/IL-17RC, ACT1, TRAF6, and TAK1 mediating IL-17-induced NF-κB/MAPK activation and neutrophil-recruiting cytokines. IL-12-driven JAK-TYK2-STAT4 signalling promotes Th1 differentiation and IFN-γ production, while IL-12Rβ1/β2 and IFNGR1/2 via JAK-STAT1 signalling control IFN-γ-dependent target responses, including interferon-stimulated genes such as IRF1, that are essential for antifungal immunity. *CARD9*: Caspase Recruitment Domain-Containing protein 9; *BCL10*: B-Cell Lymphoma/Leukemia 10; *SYK*: Spleen Tyrosine Kinase; *IL-1β*: Interleukin-1 beta; *IL-18*: Interleukin-18; *MALT1*: Mucosa-Associated Lymphoid Tissue Lymphoma Translocation Protein 1; CBM Complex: CARD-BCL10-MALT1 complex; *IKKa*: IκB Kinase alpha ; *IKKβ*: IκB Kinase beta; *IKKƔ*: IκB Kinase gamma; *NF-κB*: Nuclear Factor kappa-light-chain-enhancer of activated B cells; *JAK*: Janus kinase; *TYK2*: Tyrosine kinase 2; *STAT*: Signal Transducer and Activator of Transcription; *RORƔt*: Retinoic acid-related orphan receptor gamma t; IL17RA: Interleukin-17 receptor A; IL17RC: Interleukin-17 receptor C; *ACT1*: NF-κB activator 1; *TRAF6*: TNF receptor-associated factor 6; *TAK1*: Transforming growth factor-β-activated kinase1; IL-12RB1: Interleukin-12 Receptor Beta 1; IL-12RB2: Interleukin-12 Receptor Beta 2; IFN-Ɣ: Interferon-gamma; Th1: T helper 1; Th17: T helper 17; *IFNGR1*: Interferon-gamma receptor 1; *IFNGR2*: Interferon-gamma receptor 2; *ISGs*: Interferon-stimulated genes; *IRF1*: Interferon Regulatory Factor 1. The figure was created using BioRender

Fungi are a vital part of the human microbiota and are usually commensals. However, several fungal species, including *Pneumocystis*,* Aspergillus*,* Candida*,* Histoplasma*,* Rhizopus*, and *Cryptococcus*, are major human pathogens in immunocompromised hosts [1, 2] (Fig. 1B). Together, these organisms cause over three million deaths worldwide each year, mainly affecting people with weakened immune systems [3–5]. Significant advances in our understanding of antifungal immunity have elucidated the cellular and molecular mechanisms underlying host defence. Seminal reviews have highlighted the critical interplay between innate and adaptive immunity in controlling fungal pathogens [6]. The initial response relies on phagocytic cells, particularly neutrophils, which engulf fungi, deploy antimicrobial peptides, and release signalling molecules like interleukin (IL)-1β and tumour necrosis factor (TNF)-α, which trigger protective inflammation and recruit additional immune effector cells [7, 8] (Fig. 1A). Central to this neutrophil response is the respiratory burst, in which the NADPH oxidase complex generates reactive oxygen species for oxidative killing, complemented by non-oxidative mechanisms and the release of neutrophil extracellular traps (NETs) [9–11]. The non-redundant importance of each is exposed by the inborn errors of immunity that disrupt them, discussed below.

Beyond neutrophils, mononuclear phagocytes are also a critical component of innate antifungal defence (Fig. 1A). Mononuclear phagocytes, including monocytes and tissue-resident macrophages, contribute to fungal control through direct fungicidal activity, along with the secretion of pro-inflammatory cytokines and chemokines that orchestrate local immune responses [6, 12]. For instance, macrophage depletion studies in mice demonstrated that the loss of these cells accelerates fungal proliferation in the kidneys, liver, and spleen and significantly increases host mortality, highlighting their essential role in antifungal host defence [13]. Anatomically, alveolar macrophages provide first-line defence against inhaled *Aspergillus* conidia, *Cryptococcus neoformans*, and *Pneumocystis jirovecii* in the lung, while hepatic Kupffer cells trap disseminating *Candida* and *Histoplasma* in the portal circulation [14–18].

In this review, we examine antifungal immunity through the lens of these inborn and acquired defects. IEIs that disrupt signalling cascades rarely confine their effects to a single cell type. Because these same pathways operate in neutrophils, monocytes and structural cells, thus variants can impact antimicrobial defence and cause syndromic features. We start from innate recognition and phagocyte function, through CARD9/NF-κB, IL-17/IL-23, and IL-12/IFN-γ signalling, to transcription factors and combined immunodeficiencies (Fig. 1C-G). IEIs reveal the non-redundant roles of these pathways by linking single-gene defects to characteristic fungal phenotypes [19, 20]. The genotype-phenotype correspondences are strikingly specific. For example, defects in NADPH oxidase (CGD) predispose to invasive aspergillosis; *CARD9* deficiency to deep dermatophytosis and CNS fungal disease; IL-17 pathway defects (IL-17RA, IL-17RC, ACT1, IL-23R) to chronic mucocutaneous candidiasis (CMC); the IL-12/IFN-γ axis to dimorphic-fungal disease; *STAT3* loss-of-function to CMC with invasive aspergillosis; *STAT1* gain-of-function to refractory CMC; and *GATA2* or *IRF8* defects to disseminated histoplasmosis and other endemic mycoses [21]. These same vulnerabilities are increasingly recapitulated in previously healthy adults by neutralising anti-cytokine autoantibodies, which are acquired phenocopies of IEIs (Summarised in Box 1). With genetic diagnosis now faster and more accessible, and with pathway-targeted therapies emerging, a mechanistic understanding of these disorders carries direct clinical consequences. Finally, we translate these insights into clinical red flags, diagnostic algorithms, and mechanism-directed therapy. 

**Fig. 2:**
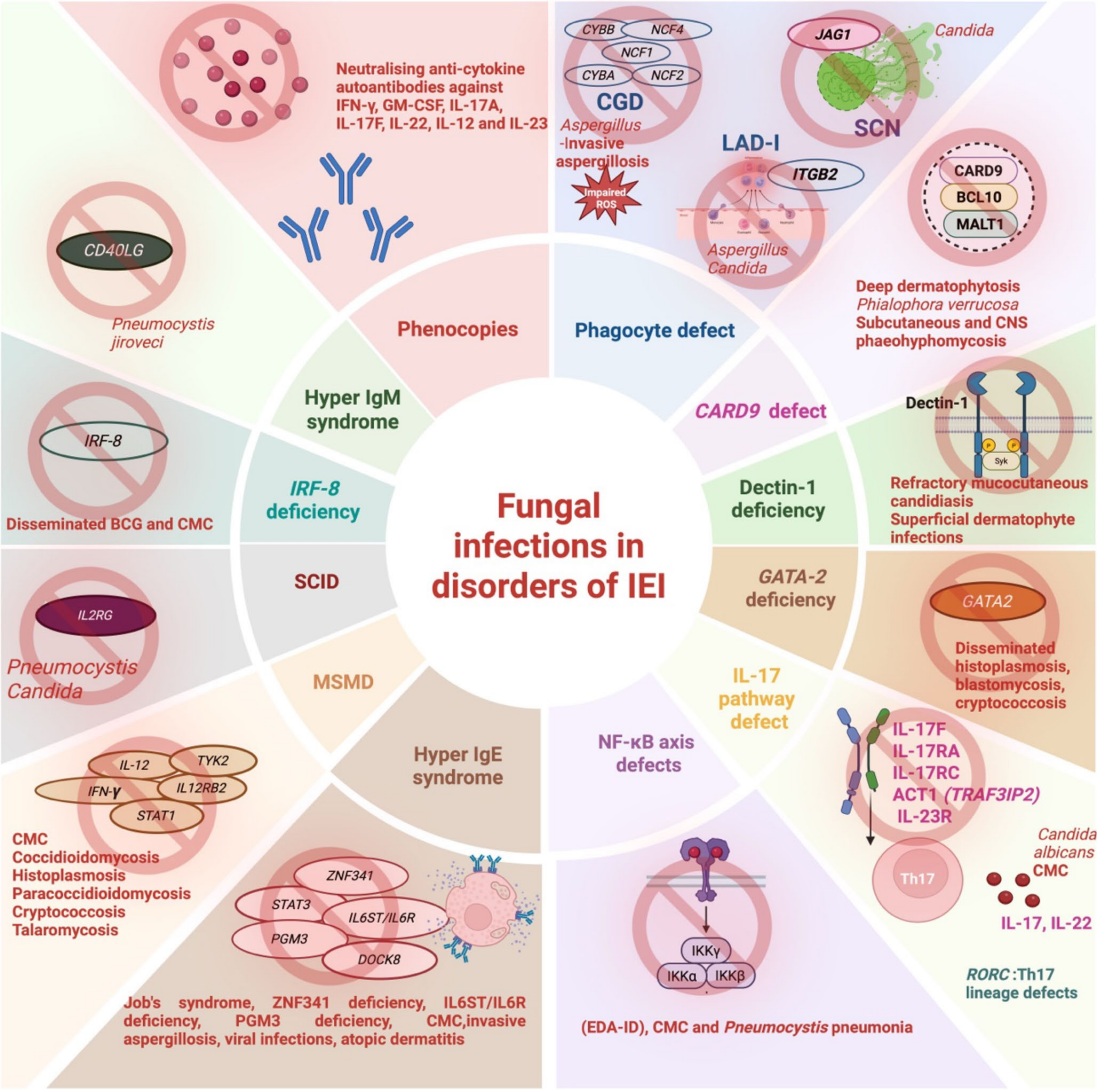
Fungal infections associated with specific inborn errors of immunity. CGD*-* Chronic granulomatous disease; *CYBB*- Cytochrome b-245 beta chain (gp91phox); *CYBA*- Cytochrome b-245 alpha chain (p22phox); *NCF1*- Neutrophil cytosolic factor 1 (p47phox); *NCF2*- Neutrophil cytosolic factor 2 (p67phox); *NCF4*- Neutrophil cytosolic factor 4 (p40phox); ROS- Reactive oxygen species; SCN- Severe congenital neutropenia; *JAGN1*- Jagged-1; LAD-I- Leukocyte adhesion deficiency type I; *ITGB2*- Integrin beta-2 (CD18); *CARD9-* Caspase Recruitment Domain-Containing protein 9; *GATA2-* GATA-binding factor 2; IL-17 F- Interleukin-17 F; IL-17R- Interleukin-17 Receptor A; IL-17RC- Interleukin-17 Receptor C; ACT1/TRAF3IP2- NF-κB Activator 1/TNF Receptor-Associated Factor 3 Interacting Protein 2; IL-23R- Interleukin-23 Receptor; *RORC-* Retinoic Acid Receptor-Related Orphan Receptor C; NF-kB- Nuclear Factor kappa-light-chain-enhancer of activated B cells; *IKKα*,* IKKβ*, and *IKKγ* stand for IκB Kinase Alpha, IκB Kinase Beta, and IκB Kinase Gamma, respectively; *STAT3*- Signal Transducer and Activator of Transcription 3; *ZNF341-* Zinc Finger Protein 341; *IL6ST-* Interleukin 6 Signal Transducer; *IL6R-* Interleukin 6 Receptor; *PGM3-* Phosphoglucomutase 3; MSMD- Mendelian susceptibility to mycobacterial disease; *IL-12*- Interleukin-12; *TYK2*- Tyrosine kinase 2; *IFNγ* - Interferon-gamma; *STAT1*- Signal Transducer and Activator of Transcription 1; *IL12RB2*- Interleukin-12 receptor subunit beta-2; SCID- Severe combined immunodeficiency; *IL2RG*- Interleukin-2 receptor subunit gamma (common γ chain); *IRF8-* Interferon Regulatory Factor 8; *CD40LG*- CD40 ligand (CD154); GM-CSF- Granulocyte-Macrophage Colony-Stimulating Factor. The figure was created using BioRender

Inborn errors of immunity (IEIs) predisposing to fungal disease can be grouped by the immune pathway disrupted, with each category producing a relatively distinct fungal phenotype (Fig. 2).

**Box 1** An overview of inborn errors of immunity predisposing to fungal infection

**Table:** 

• Phagocyte defects: chronic granulomatous disease (CGD) and severe congenital neutropenia predispose to invasive aspergillosis (notably Aspergillus nidulans in CGD) and mucormycosis; myeloperoxidase deficiency is associated with candidiasis, while leukocyte adhesion deficiency type I (LAD-I) leads to severe bacterial infections and mucocutaneous candidiasis.
**• Fungal-recognition and CARD9-pathway defects**: *CARD9* deficiency drives deep dermatophytosis, subcutaneous and CNS phaeohyphomycosis; inherited Dectin-1 *(CLEC7A)* deficiency predisposes to refractory mucocutaneous candidiasis and superficial dermatophyte infections.
**• NF-κB axis defects**: *NEMO/IKBKG* and *IKBA* deficiencies cause anhidrotic ectodermal dysplasia with immunodeficiency (EDA-ID), featuring chronic mucocutaneous candidiasis (CMC) and *Pneumocystis* pneumonia; RELB deficiency results in a combined immunodeficiency with CMC and *Pneumocystis* pneumonia but without ectodermal dysplasia.
**• IL-17 pathway defects**: IL-17 F, IL-17RA, IL-17RC, ACT1 *(TRAF3IP2)*, and IL-23R deficiencies cause isolated CMC with or without staphylococcal disease.
**• IL-12/IFN-γ axis defects**: Mendelian susceptibility to mycobacterial disease and predispose to dimorphic-fungus infection, talaromycosis, histoplasmosis, coccidioidomycosis and paracoccidioidomycosis.
**• Hyper-IgE syndromes**: *STAT3*-LOF (Job syndrome), *ZNF341* deficiency, IL6ST/IL6R deficiency, and *PGM3* deficiency present with CMC, cold staphylococcal abscesses, severe eczema, and structural lung disease with invasive aspergillosis; *DOCK8* deficiency manifests with severe viral infections, atopic dermatitis, food allergies, elevated IgE, and mucocutaneous candidiasis, but lacks the typical cold abscesses and pneumatoceles.
***• STAT1*** **gain-of-function**: Refractory CMC with autoimmunity and increasingly recognised invasive dimorphic-fungus disease.
**• Transcription factor defects**: *GATA2* (with disseminated histoplasmosis, blastomycosis, and cryptococcosis), *IRF8* (with disseminated BCG and CMC), and *RORC* (with CMC) represent monogenic dendritic cell or Th17 lineage defects.
***• APS-1/AIRE*** **deficiency**: CMC through anti-IL-17 and anti-IL-22 autoantibodies.
**• Severe combined immunodeficiencies** (X-linked SCID, ADA-SCID, and others): confer broad susceptibility, including to *Pneumocystis jirovecii* pneumonia and disseminated candidiasis.
**• Acquired phenocopies**: neutralising anti-cytokine autoantibodies against IFN-γ, GM-CSF, IL-17 A/F, IL-22, IL-12 and IL-23 – reproduce the antifungal vulnerabilities of their inborn counterparts in previously healthy adults.

The remainder of this review treats each of these categories in turn, and Fig. 2 summarises the gene-fungus associations.

## Frontline Defence in Innate Immunity: Mechanisms of Neutrophils

Phagocytes, particularly neutrophils, play a pivotal role in this frontline defence. Neutrophils are equipped with a diverse arsenal of antimicrobial molecules, such as the calprotectin complex, lysozymes and defensins, cofactor-binding proteins, neutral serine proteases, bactericidal-increasing protein, myeloperoxidase (MPO), and the NADPH oxidase system [22, 23]. Once fungal cells are internalized, they are subjected to both oxidative and non-oxidative killing mechanisms. For example, the NADPH oxidase complex, composed of subunits such as *CYBB*,* CYBA*,* NCF1*,* NCF2*, and *NCF4*, catalyses the production of superoxide radicals [24]. These radicals are rapidly dismutated into hydrogen peroxide by superoxide dismutase, and in the presence of MPO, hydrogen peroxide is further transformed into potent antimicrobial agents such as hypohalous acids [25]. This cascade not only directly damages fungal cells but also facilitates the activation of neutrophilic proteases via potassium flux, thereby augmenting intracellular fungal killing.

The consequences of disrupting this machinery are well illustrated by chronic granulomatous disease (CGD). In CGD, mutations in genes encoding NADPH oxidase subunits lead to reduced reactive oxygen species (ROS) production [26]. This deficiency compromises neutrophil-mediated fungal clearance, predisposing patients to invasive infections such as aspergillosis [27]. Beyond infectious susceptibility, CGD is paradoxically characterized by a hyperinflammatory state. Impaired fungal clearance, coupled with defective neutrophil apoptosis and efferocytosis, leads to persistent immune activation [28]. This dysregulation drives cytokine production and granuloma formation, resulting in significant inflammatory tissue damage [28]. This impacts patient outcomes despite antimicrobial therapies. Interestingly, while the oxidative killing pathway is disrupted in CGD, certain fungal species, including *Mucor*, *Candida*, and *Cryptococcus*, are less frequently detected, suggesting that non-oxidative mechanisms can, at times, partially compensate for the ROS deficit [29–31].

Neutrophils also deploy effector mechanisms beyond intracellular killing. By forming neutrophil extracellular traps (NETs), neutrophils contribute to antifungal defence [32]. Recent findings have improved our understanding of NETosis in fungal infections. For example, mutations in *JAGN1*, a gene linked to severe congenital neutropenia, have been shown to impair NET functionality despite ongoing NET release [33]. In these cases, reduced MPO expression was found to compromise the candidacidal activity of NETs, a defect that can be ameliorated by treatment with granulocyte-macrophage colony-stimulating factor (GM-CSF) or exogenous MPO [33].

Effective antifungal responses also depend on the proper migration and adhesion of immune cells. Leukocyte extravasation is orchestrated by adhesion molecules such as selectins and integrins, which facilitate the exit from the bloodstream and the arrival at infection sites [33]. Mutations in *ITGB2*, which encodes the β2 integrin subunit (CD18), cause leukocyte adhesion deficiency type I (LAD) [34, 35]. In LAD, the failure of neutrophils to reach tissues results in recurrent mucosal infections with pathogens, including *Aspergillus* and *Candida* species [36].

## Recognition of Fungi by Host Immune Cells

Mononuclear phagocytes initiate antifungal immune responses through the engagement of diverse pattern-recognition receptors (PRRs) (Fig. 1B). These PRRs, including Toll-like receptors (TLRs), C-type lectin receptors (CLRs), retinoic acid-inducible gene 1 (RIG-I)-like receptors (RLRs), and NOD-like receptors (NLRs), detect conserved fungal cell wall components such as chitin, mannans, and glucans, which are known as pathogen-associated molecular patterns (PAMPs) [37] (Fig. 1B). Recent studies have expanded our understanding of these interactions, revealing that additional receptors and co-receptors may modulate the sensitivity and specificity of fungal detection [38, 39]. Upon binding fungal PAMPs, these receptors activate downstream signalling cascades that stimulate the production and secretion of inflammatory mediators, including cytokines and chemokines, thereby priming and recruiting additional immune cells to the site of infection [40]. Amongst the PRRs, CLRs have emerged as central sensors of fungal pathogens (Fig. 1B). Following fungal sensing, CLRs, including Dectin-1, mediate phagocytosis, oxidative and non-oxidative killing, and LC3-associated phagocytosis (LAP). They signal through Syk kinase, which activates the CARD9-BCL10-MALT1 (CBM) complex, leading to NF-κB and NFAT-dependent transcriptional responses [41, 42] (Fig. 1C). In parallel, CLR-Syk signalling can engage caspase-1 activation, promoting the maturation of pro-IL-1β and pro-IL-18 via canonical inflammasome pathways [43, 44]. The defects in these signalling pathways associated with fungal diseases are detailed below.

Dendritic cells link innate and adaptive immunity by processing fungal antigens and presenting them to CD4 + helper T cells via major histocompatibility complex (MHC) II molecules [45]. They further shape adaptive immunity through the production of polarizing cytokines, including IL-12, IL-6, TGF-β, IL-1β, and IL-23, which direct the differentiation of Th1, Th17, and regulatory T-cell subsets. IL-12 promotes Th1 responses and IFN-γ production, enhancing macrophage fungicidal activity, whereas IL-1β, IL-6, TGF-β, and IL-23 support Th17 differentiation and the production of IL-17 and IL-22, which are crucial for mucosal antifungal defence and neutrophil recruitment. The subsequent adaptive immune response is primarily mediated by T-helper 1 (Th1) and T-helper 17 (Th17) responses [45, 46]. Systemic fungal dissemination is controlled via interferon (IFN)-γ production, a hallmark of Th1 immunity [45]. In contrast, Th17 responses are essential for maintaining integrity and mounting defence at mucosal surfaces [46, 47]. The protective output of this axis depends not only on Th17 generation but on intact IL-17 receptor signalling in epithelial cells. This signalling is transduced through IL-17RA/IL-17RC and the adaptor ACT1, and on the upstream STAT3- and IL-23-driven programmes. These sustain Th17 differentiation, whereas the opposing transcription factor STAT1 restrains this axis. The development of the mononuclear phagocytes and dendritic cells that initiate these responses is itself governed by myeloid transcription factors such as GATA2 and IRF8. As described in the sections that follow, inborn errors at each of these nodes - IL-17RA, IL-17RC, ACT1, IL-23R, STAT3, STAT1, GATA2 and IRF8 produce distinct fungal infection profiles. Therefore, host defence requires coordinated epithelial-barrier function, neutrophil and mononuclear phagocyte activity, fungal pattern recognition, and adaptive Th1/Th17 immunity.

CD4⁺ T-cell effector cytokines orchestrate macrophage activation, thereby influencing disease outcome. Th1-driven M1 polarisation enhances fungal killing, whereas Th2-driven M2 polarisation impairs clearance and facilitates intracellular fungal survival. This strategy is actively exploited by *C. neoformans*, which subverts macrophage function to establish a protected intracellular niche [48, 49]. Beyond *C. neoformans*, tissue-resident macrophages are the primary cells that combat intracellular dimorphic fungi, including *Histoplasma capsulatum* and *Talaromyces marneffei* [50, 51]. These pathogens have evolved specific mechanisms to survive within the phagolysosomal compartment. The functional capacity of these cells, which is shaped by their tissue of origin, developmental ontogeny, and local cytokine environment, is therefore central to host defence against this important class of fungal pathogens.

### **Fungal PAMPs and Their Cognate Receptors**

Antifungal immunity is initiated by the recognition of conserved fungal cell wall components by germline-encoded PRRs on phagocytes (Fig. 1), which are summarised in Table 1.

**Table 1:** Key PAMPs and engaging CLRs, TLRs, NLRs and RLRs are listed for each pathogen involved in mediating recognition of major human fungal pathogens

Fungal Pathogen	Key PAMPs	CLRs	TLRs	NLRs	RLRs	References
*Candida albicans*	β-glucans, α-mannans, phospholipomannan, O-linked mannans, fungal RNA/DNA, chitin	Dectin-1 (β-glucans), Dectin-2 & Mincle (α-mannans), MR & DC-SIGN (mannose)	TLR2 (phospholipomannan), TLR4 (O-linked mannans), TLR7 (RNA), TLR9 (CpG DNA)	NLRP3 (hyphae-induced IL-1β), NLRC4 (mucosal defence), NLRP4, NLRP10	MDA5 (hyphae-induced IFN-β)	[52–59]
*Aspergillus fumigatus*	β-glucans (conidia), α-mannans, fungal DNA, chitin	Dectin-1 (β-glucans, conidia), Dectin-2 & Mincle (mannans), MR	TLR2 & TLR4 (stage-dependent recognition of conidia; signalling altered upon germination); TLR9 (CpG DNA)	NLRP3 & AIM2 (IL-1β & IL-18), NOD1 & NOD2 (cytokine release)	-	[60–65]
*Cryptococcus neoformans*	Capsular GXM (glucuronoxylomannan), mannose residues, β-glucans (masked by capsule)	Dectin-2 (mannans), MR (mannose residues)	TLR2, TLR4, TLR9	NLRP3 (IL-1β)	-	[66–69]
*Histoplasma capsulatum*	Mannans, β-glucans	Dectin-2 (primary; activates NLRP3), MR (mannose), Dectin-1 (secondary; limited due to α-glucan masking of β-glucan)	TLR2, TLR4	NLRP3 (IL-1β via Dectin-2)	-	[70]
*Pneumocystis jirovecii*	β-glucans, α-mannose, phospholipomannan	Dectin-1 (β-glucans), Mincle (glycolipid ligands), Dectin-2	TLR2	NLRP3	-	[71]
*Rhizopus* species	β-glucans, mannans, spore-surface ligands	Dectin-1 (β-glucans, sporangiospores), Dectin-2 (mannans), MR (mannose residues)	TLR2, TLR4 (spore recognition), TLR9 (fungal DNA)	NLRP3 (IL-1β), NOD2	-	[72]
*Malassezia* species	α-mannose (hydrophilic), lipophilic ligands	Dectin-2 (hydrophilic mannans), Mincle (lipophilic ligands)	TLR2	NLRP3 (IL-1β via Syk)	-	[73, 74]

MR =Mannose Receptor; DC-SIGN = Dendritic Cell-Specific ICAM-3-Grabbing Non-Integrin; CLR = C-type Lectin Receptor; TLR = Toll-like Receptor; NLR = NOD-like Receptor; RLR = RIG-I-like Receptor; PAMP = Pathogen-Associated Molecular Pattern

## CARD9 and CLR-mediated pathways

As mentioned above, fungi are predominantly recognised by CLRs, which signal via CARD9, thereby activating canonical NF-κB signalling [75] (Fig. 1). Mutations in this pathway have been linked to systemic and mucosal fungal infections [76–81]. The association between genetic defects and an increased risk of CMC, dermatophytosis, and fungal meningitis was demonstrated in a consanguineous Iranian family with a homozygous point mutation, p.Q295X, in *CARD9* [82]. Recent studies have identified additional CARD9 mutations, such as p.G72S and p.R373P, that are associated with CMC and *Candida*-related meningitis [83]. It is important to note that in over 40% of patients with *CARD9* deficiency who present with *Candida* infections, the majority have central nervous system (CNS) candidiasis [82, 83]. In addition to CNS candidiasis, *CARD9* deficiency has been observed in patients with extrapulmonary *Aspergillus* infections, despite the absence of pulmonary aspergillosis [84]. Interestingly, these patients exhibit normal neutrophil responses to *A. fumigatu*s [84, 85]. Recent research identified two novel homozygous *CARD9* mutations, p.Q289X and p.R101C, in 17 patients from Morocco, Tunisia, and Algeria who developed severe deep-seated dermatophytosis [79]. Furthermore, four unrelated Chinese patients developed severe subcutaneous phaeohyphomycosis caused by the fungus *Phialophora verrucosa*, carrying novel point and frameshift mutations in CARD9 that impaired Th17-mediated antifungal immunity [78]. Mechanistically, this may be because CARD9 abnormalities reduce cytokine production, such as IL-6 and IL-1β, which, in turn, leads to Th17 cell deficiencies and ultimately predisposes patients to *Phialophora verrucosa* infections. Notably, candidiasis was not observed in these patients [78], nor in most patients with the p.Q289X and p.R101C mutations [79]. In both humans and mice, CARD9 deficiency results in impaired phagocytic effector functions, decreased production of pro-inflammatory cytokines and chemokines (including IL-6, IL-1β, and CXCL1), and aberrant CD4 T cell responses [76, 86, 87]. CARD9 also plays an important role in regulating fungal communities in the gastrointestinal tract (GI) [88]. CARD9 deficiency leads to aggravated inflammatory bowel disease (IBD) mediated by *Malassezia* overgrowth, which is usually considered a commensal fungus [88].

Key aspects of human anti-cryptococcal immunity remain poorly defined among individuals with apparently intact T-cell immunity. The protective immune response to *Cryptococcus neoformans*, a World Health Organisation fungal priority pathogen, is centred on Th1-driven IFN-γ production and the activation of lung macrophages into a classical (M1) phenotype, which produces nitric oxide and reactive oxygen intermediates that restrict yeast growth [89, 90]. Experimental mouse studies demonstrate that CARD9 signalling is crucial for the Th1/M1 axis: CARD9-deficient macrophages skew towards an M2 phenotype, which is conducive to cryptococcal survival [91, 92]. However, direct human validation remains elusive. In humans, documented CARD9 mutations are rare, and most clinical cryptococcal cases occur in the context of profound T cell or IFN-γ pathway defects (e.g., HIV infection, solid organ transplant). Also, human CARD9-deficiency cohorts are so far small, and because cryptococcal disease typically manifests in severely immunocompromised patients, the specific signature of impaired M1 macrophage activation via CARD9 has not been clearly demonstrated in human cryptococcosis [83, 91]. As a result, while the mouse data strongly implicate CARD9 in the Th1/M1 antifungal axis, the paucity of human cases and the dominance of other immunodeficiency contexts mean that we do not yet see a well‐defined human syndrome of CARD9-mediated failure of M1 macrophage anti-*Cryptococcus* immunity. Notwithstanding, a recent outbreak of cryptococcosis in China among 251 HIV-negative individuals was linked to defective Dectin-2 (which signals via CARD9) and was limited to severe pulmonary disease, without CNS involvement [69]. Conversely, other pattern recognition pathways appear redundant. TLR signalling demonstrates limited impact on susceptibility to cryptococcosis. Nakamura et al. reported that Dectin-1 is not essential for effective immune defence against *Cryptococcus neoformans* [93]. *Cryptococcus* may evade Dectin-1 detection because its polysaccharide capsule effectively masks β-glucan ligands. This evasion strategy shifts immune reliance to alternative receptors, such as Dectin-2 and CARD9-dependent pathways, in human cryptococcal defence [93].

### **Inherited Dectin-1 Deficiency**

Dectin-1, the principal β-glucan receptor encoded by *CLEC7A*, is the dominant innate sensor for fungal cell wall components in monocytes, macrophages, and dendritic cells. Inherited deficiency of this receptor arises most commonly from the stop-gain variant Y238X (c.714T > G; rs16910526). This truncates the C-terminal carbohydrate-recognition domain, impairing both surface trafficking and β-glucan binding [94]. A gene-dose effect is evident: homozygotes show attenuated IL-1β, IL-6 and TNF production, and heterozygotes show intermediate impairment. Clinically, the variant has been linked to recurrent mucocutaneous *Candida* infection, oesophageal candidiasis, persistent *Candida* colonisation following haematopoietic transplant, and increased susceptibility to invasive aspergillosis in haematopoietic transplant recipients [95, 96]. Unlike CARD9 deficiency, which is a Mendelian disorder, Y238X confers susceptibility with partial penetrance. The role of Dectin-1 differs between cell types; monocytes, macrophages and dendritic cells employ it as the dominant β-glucan sensor. In contrast, human neutrophils rely more heavily on complement receptor 3 (CR3/Mac-1; CD11b/CD18), which binds both iC3b-opsonised particles and β-glucan directly via its lectin-like domain, with CARD9-dependent signalling contributing predominantly to cytokine production rather than candidacidal effector activity [97, 98].

## NF-κB Activation and Its Regulation during Fungal Infection

Nuclear factor kappa B (NF-κB) represents a family of transcription factors critical for immune responses, inflammation, and cellular differentiation programs [99–102]. Because of its central role, NF-κB is tightly regulated; dysregulation can lead to aberrant inflammation and cancer. NF-κB typically exists as dimers composed of subunits such as p50, p52, REL-A (p65), REL-B, and c-REL, which are maintained in an inactive state in the cytoplasm through sequestration by IκB proteins [103–106]. Upon stimulation with antigens or cytokines, IκB is phosphorylated and degraded, allowing NF-κB to translocate to the nucleus and drive gene expression [107]. There are two primary activation pathways: the canonical pathway, regulated by IKKβ and NEMO, which is key for the canonical pathway and cell survival [108, 109], and the non-canonical pathway, regulated by IKKα [110] (Fig. 1C). Hypermorphic mutations in *IKBA* [111] and hypomorphic mutations in *IKBKG/NEMO* [112–114] are associated with Anhidrotic Ectodermal Dysplasia with Immunodeficiency (EDA-ID). The hallmark of EDA-ID is a combined immunodeficiency and ectodermal dysplasia, arising from impaired NF-κB activation. Hypomorphic *NEMO* mutations impair the recruitment and activation of the IκB kinase (IKK) complex, thereby reducing NF-κB nuclear translocation. In contrast, hypermorphic or gain-of-function mutations in *IκBα* (IKBA) stabilise IκBα and prevent its phosphorylation-induced degradation, likewise impairing NF-κB activation. Together, these mutations converge on defective NF-κB signalling [111]. Since NF-κB is downstream of receptors involved in TCR, BCR, TLR, IL-1R, IL-18R, and TNFR signalling [112–114], deficiencies in IKKα and IKBKG/NEMO manifest as complex immunodeficiencies. Notably, patients with EDA-ID not only experience recurrent invasive bacterial infections but also exhibit a marked susceptibility to fungal infections, most commonly presenting with CMC and *Pneumocystis jirovecii* pneumonia [112, 115].

### Inherited RelB Deficiency

A more recent addition to the NF-κB axis of IEIs is autosomal-recessive RELB deficiency [116]. Biallelic loss-of-function variants in *RELB*, which encode a key subunit of the non-canonical NF-κB pathway, produce a combined immunodeficiency with susceptibility to viral, bacterial, and fungal infections. Strikingly, these patients also develop secondary neutralising autoantibodies against type I interferons. This is a consequence of failed thymic stromal AIRE-driven negative selection (the non-canonical NF-κB pathway is essential for thymic medullary epithelial-cell development). RELB deficiency thus exemplifies a novel paradigm in which a single inborn defect produces both a genetic immunodeficiency and an acquired anti-cytokine phenocopy in the same patient. This collapses the previously compartmentalised categories of monogenic IEI and autoantibody-mediated disease.

## IL-17/IL-23 Axis - Signalling and Role in Antifungal Defence

The key role of the IL-17 pathway in defence against *Candida albicans* is underscored by discoveries of genetic deficiencies that collectively explain what was hitherto considered idiopathic CMC [46, 117]. Notable deficiencies include autosomal dominant (AD) IL-17 F and AR deficiencies involving IL-17 receptor A (IL-17RA), IL-17RC, and NF-κB activator 1 (ACT1) [118–121] (Figure D, E). The nonredundant role of IL-17 signalling in mucosal antifungal immunity was elegantly demonstrated in two independent kindreds with CMC. In a consanguineous family from Morocco, a homozygous nonsense mutation in *IL17RA* (c.850 C > T; p.Q284X) resulted in complete loss of IL-17RA expression and was linked to autosomal recessive CMC. Affected individuals presented with recurrent *Candida* infections of the skin and mucosa, accompanied by *Staphylococcus aureus* dermatitis and early-life respiratory infections. Despite normal frequencies of circulating IL-17/IL-22-producing Th17 cells, epithelial cells and fibroblasts failed to respond to IL-17 A or IL-17 F stimulation, demonstrating that intact IL-17 receptor signalling, rather than Th17 cell generation per se, is essential for antifungal immunity at mucosal sites [122]. In second kindred from Argentina, a heterozygous c.284 C > T, p.S65L missense variant in *IL-17 F* was linked with AD CMC [122]. This variant was accompanied by upper respiratory infections, asthma, and furunculosis. Although patients displayed normal proportions of IL-17/IL-22-producing Th17 cells and produced IL-17 F homodimers and IL-17 A/IL-17 F heterodimers, the mutant *IL-17 F* (S65L) exhibited impaired binding to IL-17RA. This dominant-negative effect resulted in decreased cytokine production by leukocytes, fibroblasts, and keratinocytes, with incomplete clinical penetrance as evidenced by two family members harbouring the same mutation who did not develop CMC [122].

Furthermore, ACT1, a critical cytoplasmic adaptor protein downstream of IL-17 receptors, has been directly implicated in mucocutaneous antifungal immunity. ACT1 (also known as CIKS) links IL-17 receptor signalling to downstream activation of NF-κB and MAPK pathways and is indispensable for IL-17-mediated host defence at epithelial surfaces [123]. In humans, a homozygous missense mutation (c.1607 C > T, p.T536I) in the SEFIR domain of *ACT1* was shown to abolish IL-17 signalling, causing AR CMC (80). The same mutation in *ACT1*, identified in two siblings from a consanguineous Algerian family, was shown to impair ACT1 recruitment to the IL-17RA/RC complex without affecting ACT1 homodimerization [124]. Fibroblasts from affected individuals failed to respond to IL-17 A and IL-17 F, while their T cells were unresponsive to IL-17E (IL-25), demonstrating the non-redundant requirement of ACT1 for both IL-17 A/F-driven inflammation and IL-17E-dependent epithelial immunity. Crucially, IL-25 (IL-17E), while structurally related to IL-17 A/F, has distinct functions as a key alarmin produced by epithelial cells that amplifies type 2 immune responses, thereby influencing mucosal barrier integrity and repair. It is worth mentioning that co-bacterial infections, particularly with *Staphylococcus aureus*, have been reported in patients with AR IL-17RA, AR IL-17 F, or ACT1 (TRAF3IP2) deficiencies, but are notably absent in individuals with AR IL-17RC deficiency. This distinction suggests that IL-17RA-dependent signalling plays a dominant role in antibacterial defence, whereas IL-17RC-associated pathways are more narrowly linked to antifungal immunity [125, 126]. IL-17RA is crucial for the signalling of IL-17 A, IL-17 F, and IL-25, while IL-17RC is specific to IL-17 A and IL-17 F. The absence of staphylococcal infections in IL-17RC-deficient patients suggests that IL-25 signalling may influence susceptibility [126]. Murine models of oropharyngeal candidiasis further highlight the importance of this pathway: IL-17RA- and IL-17RC-deficient mice, as well as mice treated with neutralizing antibodies against IL-17 A/IL-17 F, exhibit significantly higher oral *Candida* burdens compared to control groups [127, 128]. These models demonstrate that although granulocyte colony-stimulating factor (G-CSF) production, CXC-chemokine responses, and neutrophil recruitment remain intact, defective IL-17 signalling nonetheless compromises the antifungal function of key effector cells. Indeed, epithelial cells in the oral mucosa have emerged as central mediators of protection against oral candidiasis [129]. Together, these findings emphasize that genetic defects disrupting IL-17 signalling and its cross-talk with other cytokines critically impair mucosal antifungal immunity. A better understanding of these molecular interactions will not only elucidate the pathogenesis of CMC but also pave the way for targeted therapeutic interventions in patients with IEI.

### **A Novel IL-17RC Duplication Variant**

Noma et al. described a homozygous *IL17RC* duplication (c.1370_1371dup) in a Japanese child with isolated CMC and no staphylococcal disease [130]. This phenotype reinforces a well-recognised clinical dissociation: IL-17RA/ACT1 signalling protects against both *Candida* and *Staphylococcus*, whereas IL-17RC is selectively required for antifungal mucosal defence. Absence of staphylococcal infection in IL-17RC-deficient patients suggests a particular dependence on the IL-17 A/F-IL-17RA/RC axis for mucocutaneous fungal defence. This genotype thus serves as a useful natural experiment in cytokine-receptor pairing specificity.

### **Homozygous IL-23R p.R381Q Deficiency**

A recent report has added IL-23R deficiency to the IL-17/IL-23 axis of IEIs [131]. The IL-23R p.R381Q variant is common in the general population, and a single copy is protective against inflammatory bowel disease and ankylosing spondylitis. However, carrying two copies of IL-23 severely impairs IL-23-driven STAT3 phosphorylation and Th17 cytokine production, thereby causing CMC. This dose-dependent effect illustrates a key principle: one hypomorphic allele reduces harmful Th17-driven inflammation, whereas two copies abolish the protective Th17 response needed to defend against *Candida*. The same logic applies clinically. Anti-IL-23 biologics used for psoriasis or IBD can cause mucocutaneous candidiasis by pharmacologically reproducing this biallelic deficiency state.

## IL-12/IFN-γ Axis in Antifungal Defence

Type 1 immunity orchestrated by the IL-12/IFN-γ axis is essential for antifungal immunity [132] (Figure F, G). Upon pathogen encounter, dendritic cells produce IL-12p70-a heterodimer composed of IL-12p40 and IL-12p35 that is essential for driving Th1 differentiation and subsequent IFN-γ production by T cells [133]. IL-12 signals through its receptor, a heterodimer of IL-12Rβ1 and IL-12Rβ2, while IFN-γ, produced by Th1 and natural killer (NK) cells, induces interferon-stimulated genes (ISGs), including those for MHC-I and MHC-II, thereby facilitating critical crosstalk between antigen-presenting cells and the adaptive immune system [134, 135] (Figure F, G). Since 1996, it has been recognized that mutations in the IL-12/IFN-γ axis cause Mendelian Susceptibility to Mycobacterial Disease (MSMD) [135]. Mutations affecting *IL-12*, *IFN-γ* receptors, *STAT1*, *TYK2*, gp91phox, and *NEMO* not only increase susceptibility to mycobacteria but also predispose patients to infections by dimorphic fungi, non-tuberculous mycobacteria, *Salmonella*, and viruses [135, 136]. Patients with homozygous null mutations in *IFN-γR1 or IFN-γR2*, who are unable to mount effective IFN-γ responses, are particularly susceptible to disseminated infections with endemic dimorphic fungi, such as *Histoplasma capsulatum* [137, 138]. Moreover, missense mutations in the *IL-12Rβ2* subunit have been associated with infections such as coccidioidomycosis, histoplasmosis, paracoccidioidomycosis, and cryptococcosis [139–142]. Defects in the IFN-γ receptor have similarly been linked to coccidioidomycosis and histoplasmosis [138, 143, 144]. Additionally, mutations in *IL-12Rβ1*, which is shared by the IL-12 and IL-23 receptors, often result in CMC due to impaired Th1 and Th17 immunity [135, 142]. Persistent candidemia has even been linked to polymorphisms in the *IL-12p40* gene [145]. The convergence of antifungal and antimycobacterial immunity on the IL-12/IFN-γ axis underscores the need to examine their interplay in the context of IEI.

Emerging evidence suggests that co-infections involving fungi and mycobacteria are more common than usually appreciated and represent a significant clinical challenge in individuals with IEIs [146, 147]. These overlapping susceptibilities reflect a shared dependence on Th1-driven macrophage activation for effective pathogen control. In co-infection contexts, mycobacterial infection may reprogram macrophage responses through sustained IFN-γ exposure, IL-10 induction, or metabolic exhaustion, thereby impairing antifungal effector functions. Conversely, fungal infection may disrupt the IL-12/IFN-γ circuit by promoting Th2 or Th17 polarization, attenuating macrophage microbicidal activity. This pathological cross-talk suggest that susceptibility to a pathogen may be defined not merely by the absence of function but also by a dynamic state defined by immune dysregulation (Discussed in Box 5).

## Combined Immunodeficiencies with Fungal Infections

Although the disorders in this section arise from distinct molecular defects, they share a common consequence. These disorders share a common consequence, global or near-global failure of lymphocyte development, survival, or activation. This renders the host broadly susceptible to opportunistic fungi, namely, *Pneumocystis jirovecii* and *Candida* spp., but also dimorphic and encapsulated fungi in disorders where T cell-macrophage collaboration is specifically disrupted.

### X-linked and AR Hyper-IgM Syndromes (CD40L and CD40 Deficiency)

Defects in the CD40L-CD40 interaction, as in X-linked CD40L deficiency (HIGM1) and AR CD40 deficiency (HIGM3), impair T-cell help to B cells, germinal-centre formation, and class-switch recombination, producing the classical Hyper-IgM phenotype of elevated IgM with low IgG, IgA, and IgE. The fungal phenotype is dominated by *Pneumocystis jirovecii* pneumonia (often the presenting illness in infancy), *Cryptosporidium parvum*-driven sclerosing cholangitis, and increasingly recognised disseminated *Cryptococcus neoformans* infection [148–150]. Importantly, in a multicentre paediatric cohort of 50 IEI patients with *Talaromyces marneffei* infection, X-linked Hyper-IgM accounted for ~ 30% of cases, alongside STAT3-LOF (~ 20%), STAT1 GOF (~ 20%) and defects in IL-12/IFN-γ signalling and *CARD9* [151]. This highlighted that the CD40L-CD40 axis is also essential for control of dimorphic intracellular fungi [151, 152].

### IL-2 Common γ-chain

A more severe form of combined immunodeficiency arises from mutations in IL-2 receptor subunit γ (IL-2RG), also known as the common γ-chain. IL-2RG is a critical component of the receptor complexes for multiple cytokines, including IL-2, IL-4, IL-7, IL-9, IL-15, and IL-21 [153]. These cytokines govern various cellular functions, including differentiation, maturation, and survival of lymphocytes. Mutations in *IL-2RG* result in X-linked severe combined immunodeficiency (SCID), characterized by the absence of T and NK cells and the presence of non-functional B cells [154]. Consequently, patients with SCID are prone to severe, recurrent bacterial, viral, and fungal infections. In these individuals, common fungal pathogens include *P. jiroveci* and *Candida* spp [155]. Thus, patients with SCID illustrate how global lymphocyte deficiency, rather than any pathway-specific defect, creates broad susceptibility to opportunistic fungi, including *P. jirovecii* and *Candida* spp [156].

### STK4/MST1 as an Emerging Signalling Pathway in Fungal Immunity

A molecularly distinct but phenotypically overlapping combined immunodeficiency results from loss-of-function mutations in STK4/MST1. Serine-threonine protein kinase 4 (STK4), also known as MST1, encodes a 63 kDa protein involved in critical immune processes, including cell proliferation, apoptosis, and cytoskeletal rearrangements [157–159]. MST1 functions as a signalling intermediate in chemokine signalling, crucial for the activation of LFA-1 integrins and subsequent cell polarization [160, 161]. Acting downstream of Rap1, MST1 forms a complex with RAPL and Rap1, which facilitates LFA-1 integrin polarization to the cell edge, essential for the formation of the immunological synapse and leukocyte migration through the endothelium [160, 162–165]. Autosomal recessive (AR) nonsense mutations in *MST1* result in immunodeficiencies with a common variable immunodeficiency (CVID)-like phenotype [163]. Notably, CMC has been observed in some patients with MST1 deficiency (86), likely due to reduced T lymphocyte numbers and enhanced FAS-induced apoptosis of naïve T cells [163, 166]. Recent research has unveiled a novel pathway in which MST1 regulates epidermal innate immunity through DAF-16, an ortholog of the mammalian Forkhead box O (FOXO) transcription factor [167–170]. Although demonstrated to date largely in invertebrate models, this pathway involves calcium release and ROS production via BLI-3 dual-oxidase, activating FOXO/DAF-16 through MST1/CST-1 in *Caenorhabditis elegans* [168]. These findings provide new insights into the role of MST1 in controlling fungal infections. While STK4/MST1 deficiency is recognised as causing combined immunodeficiency in humans, data on its impact specifically on antifungal immunity remains poorly characterised. No published human studies have specifically characterised fungal-specific Th17, macrophage or neutrophil responses in STK4/MST1-deficient patients; mechanistic insight remains confined to murine models [162, 163].

## Transcription Factors Involved in Antifungal Immunity

Signal Transducers and Activators of Transcription (STATs) are cytoplasmic transcription factors involved in signal transduction of various molecules, including cytokines (IFN-γ, IL-6, IL-12, IL-23, and IL-27) [171]. Historically, interferons were regarded primarily as antiviral proteins, but it is now established that STAT-dependent interferon signalling also governs apoptosis, inflammation, and adaptive immune polarisation [172–175]. The non-redundant importance of individual STATs in fungal defence is demonstrated by the distinct infection phenotypes that arise when specific family members are disrupted, as detailed below [176]. AD gain-of-function (GOF) mutations in *STAT1* have been identified in approximately half of the patients with CMC [177]. These mutations occur in the coiled-coil domains or DNA-binding motifs and result in aberrant nuclear dephosphorylation of activated STAT1, leading to subsequent cytosolic hyperphosphorylation [178]. Mouse models harbouring these mutations have demonstrated impaired RORγt-driven Th17 differentiation, a key mediator of CMC susceptibility [179]. Enhanced responsiveness to STAT1-coupling cytokines such as IFN-γ and IL-27 hinders Th17 development, primarily through STAT1-mediated antagonism of the STAT3/RORγt axis and, in the case of IL-27, by inducing IL-10 [180, 181]. Mouse models with *STAT1* GOF mutations further confirm that increased sensitivity to these cytokines exacerbates the suppression of Th17 cells, elucidating the molecular basis of fungal susceptibility [182]. Additionally, deleterious biallelic mutations in the retinoic acid receptor-related orphan receptor C (*RORC*), which encodes RORγ and RORγt, have been reported in patients with mild CMC [183]. Mouse models with *RORC* mutations or deletions show impaired Th17 cell differentiation and increased susceptibility to fungi [183]. Wang et al. identified a c.604 A > G; p.M202V mutation in STAT1 in a case of refractory fusariosis [184]. This is consistent with the broader recognition that STAT1 GOF predisposes to invasive fungal infections beyond CMC, including disseminated dimorphic fungal disease. Mouse models with similar *STAT1* mutations have provided insights into antifungal resistance mechanisms [185]. Severe disseminated coccidioidomycosis and histoplasmosis have been observed in these patients, and corresponding mouse models have advanced our understanding of the underlying pathophysiology [186, 187]. We highlight recent advances in STAT1 research in Box 2.

**Box 2** New insights: STAT1

**Table:** 

Recent mechanistic studies (2023–2024) have revealed that STAT1 GOF mutations not only enhance STAT1 phosphorylation but also induce profound changes in chromatin remodelling and epigenetic modifications [188–190]. High-resolution ChIP-seq analyses have shown that these mutations lead to abnormal histone modification patterns at the promoters of key TH17-related genes (e.g., IL-17 A and IL-17 F), reducing the accessibility of transcriptional machinery and thereby impairing antifungal cytokine production [191].
Additionally, updated clinical cohort analyses have expanded the clinical spectrum associated with STAT1 GOF mutations, demonstrating that these patients can present with disseminated fungal infections, including coccidioidomycosis and histoplasmosis, in addition to CMC [192, 193]. These observations suggest that both genetic and epigenetic factors contribute to the variability in clinical outcomes.

In contrast, patients with AD hyper-IgE syndrome (HIES), or Job’s syndrome, exhibit defective STAT3 signalling. This defect primarily manifests as impaired Th17 differentiation, which is critical for mucosal antifungal immunity [194, 195]. Th17 cells, which express chemokine receptors such as CCR6 and CCR4 and produce IL-17 and IL-22, are essential for maintaining epithelial barrier integrity [196]. HIES patients, who typically carry heterozygous missense mutations or in-frame deletions in *STAT3*, often present with features like skeletal abnormalities, eczema, and increased susceptibility to bacterial infections, in addition to chronic CMC and dermatophyte infections [197, 198]. Pulmonary aspergillosis is also observed, particularly in the context of structural lung damage from recurrent bacterial pneumonia [199]. Invasive and extrapulmonary aspergillosis, as well as histoplasmosis, have been linked to *STAT3* mutations, underscoring its role in both systemic and mucosal antifungal defence [199]. Notably, there is clinical overlap between STAT3 deficiency and DOCK8 (guanine nucleotide exchange factor critical for cytoskeletal remodelling and immune synapse formation) deficiency in patients with hyper-IgE phenotypes [200]. DOCK8 mediates STAT3 nuclear translocation required for transcription of STAT3-dependent genes governing Th17 differentiation [201]. This evidence further provides a mechanistic link between these two disorders; DOCK8 deficiency is discussed in detail below (Box 3).

**Box 3** New Insights: STAT3 and DOCK8

**Table:** 

Emerging studies have broadened our understanding of STAT3 by demonstrating its role in maintaining epithelial barrier integrity. Recent transcriptomic and imaging studies (2023–2024) of lung epithelial cells have revealed that *STAT3* mutations are associated with the downregulation of genes encoding tight junction proteins and mucins, thereby compromising mucosal barrier function and facilitating fungal invasion [202]. Furthermore, new research has elucidated the interplay between DOCK8 and STAT3 [200].
Proteomic analyses have identified additional molecular partners that promote STAT3 nuclear translocation [203]. This could explain why patients with *DOCK8* deficiency often exhibit features overlapping with those of *STAT3* mutations [200]. These insights deepen our understanding of STAT3’s multifaceted role and point to potential therapeutic targets to restore both immune signalling and barrier integrity.

## GATA2, IRF8, and Other Key Transcription Factors

Among the transcription-factor defects, GATA2 deficiency stands out for its striking susceptibility to disseminated fungal disease. GATA2 is essential for myeloid cell differentiation, and a spectrum of mutations, including deletions, missense mutations, and frameshift mutations, leads to defective GATA2 mRNA, resulting in clinical manifestations such as B-cell, monocyte, and dendritic cell deficiencies, bone marrow hypocellularity, and myeloid dysplasia [204]. These clinical manifestations, collectively termed MonoMAC syndrome, are increasingly recognised for their marked susceptibility to systemic fungal infections, including those caused by *Histoplasma capsulatum*, *Aspergillus* spp., and *Cryptococcus neoformans*. Approximately one-third of affected individuals develop invasive fungal disease, underscoring the critical role of impaired monocyte and dendritic cell function in antifungal defence. Although these patients also exhibit vulnerability to mycobacterial infections, papillomaviruses, and myelodysplasia, the prominence of fungal infections highlights the indispensable role of mononuclear phagocytes in coordinating protective immunity against fungal pathogens [205]. Interestingly, GATA2 deficiency is the only monogenic condition reported to be associated with blastomycosis [206]. We report recent insights on GATA2 in Box 4.

Interferon regulatory factor 8 (IRF8) is another critical transcription factor required for the differentiation of myeloid progenitors into monocytes. AR mutations in *IRF8*, such as the K108E defect, result in a severe form of MSMD and combined immunodeficiency, characterised by a complete loss of circulating monocytes and dendritic cells [207]. Conversely, milder mutations, such as T80A, lead to selective depletion of conventional dendritic cells (cDC1) and have been associated with CMC and recurrent disseminated Bacillus Calmette-Guérin (BCG) disease following vaccination [208]. Functional studies have shown that cells lacking IRF8 exhibit reduced expression of IL-12, IFN-γ, TNF, IL-10, and IL-6, although IL-12 treatment partially restores IFN-γ expression [209].

**Box 4** New Insights GATA2 and IRF8

**Table:** 

Cutting-edge single-cell RNA sequencing (scRNA-seq) studies have recently been applied to patient samples with GATA2 and IRF8 deficiencies [210, 211]. These analyses have uncovered a previously unappreciated heterogeneity within monocyte and dendritic cell populations. Specific subpopulations show distinct transcriptional profiles with variable expression of antifungal effector molecules and cytokine receptors, correlating with the degree of susceptibility to fungal infections.
For IRF8, new data indicate that even partial loss of function can result in markedly decreased production of key cytokines such as IL-12 and IFN-γ, thereby impairing effective antifungal responses [209]. These findings provide a nuanced understanding of how defects in myeloid transcriptional regulators contribute to the overall immunodeficient state.

## Bridging Innate and Adaptive Immunity - The Multifaceted Role of DOCK8

DOCK8, a guanine nucleotide exchange factor, is critical for cytoskeletal rearrangement, immune cell migration, immunological synapse formation, and effective antigen presentation [212]. Recent studies have demonstrated that impaired DOCK8 function disrupts the development and maintenance of Th17 cells, which are essential for mucosal defence against *Candida* and other fungal pathogens [213]. Among IEIs, DOCK8 deficiency offers a unique window into fungal-viral co-infections. Alongside recurrent mucocutaneous fungal infections, patients with DOCK8 mutations exhibit combined immunodeficiency characterized by chronic viral infections (HPV, HSV, molluscum contagiosum, EBV) [212]. Mechanistically, DOCK8 regulates actin cytoskeletal remodelling, which is essential for immune synapse formation, lymphocyte migration, and Th17 differentiation. DOCK8 deficiency, therefore, impairs both cytotoxic antiviral responses and IL-17-mediated antifungal defence, leading to a dual breakdown in epithelial barrier integrity and immune coordination. Chronic viral persistence in the skin and mucosa further predisposes to secondary fungal invasion, mirroring patterns seen in acquired states such as HIV-Cryptococcus and COVID-19-associated *Aspergillus* infections. Thus, DOCK8 deficiency underscores how a single-gene defect disrupting cytoskeletal and signalling crosstalk can reveal shared immune dependencies that govern resistance to both viral and fungal pathogens.

## Infection Susceptibility Due to Cytokine Autoantibodies: Phenocopies of Inborn Errors of Immunity

An important development in clinical immunology is the discovery of anti-cytokine autoantibodies (ACAAs) that cause adult-onset immunodeficiency syndromes mimicking monogenic defects [214]. These neutralising autoantibodies against key cytokines result in acquired phenocopies of IEIs, effectively silencing critical immune pathways [215]. The clinical presentation is very striking and emphasises the non-redundant role of the target cytokine. For example, autoantibodies against IFN-γ are found in infections with *Talaromyces marneffei* and histoplasmosis, while autoantibodies to granulocyte-macrophage colony-stimulating factor (GM-CSF) are linked to *Cryptococcus gattii* infections [216]. Additionally, CMC is associated with neutralising autoantibodies against IL-17 A, IL-17 F and IL-22, most characteristically in APS-1. As discussed above, loss of AIRE-dependent thymic tolerance results in the production of these anti-cytokine antibodies, alongside the aberrant type 1 mucosal inflammation that drives candidiasis [214, 217]. Whether additional mechanisms, including aberrant IFN-γ/STAT1 activity at mucosal surfaces, contribute independently to fungal susceptibility in APS-1 remains debated. Patients without detectable anti-cytokine autoantibodies also develop CMC, suggesting the full picture is not yet resolved. AIRE deficiency and IFN-γR deficiency illustrate complementary aspects of the IL-12/IFN-γ axis. Loss of IFN-γ signalling predisposes to disseminated infection with intracellular pathogens and endemic fungi, while dysregulated IFN-γ/STAT1 activity in the context of impaired Th17 cytokine availability may exacerbate mucosal vulnerability. Single-gene defects that disrupt antifungal cytokine signalling (including IFN-γ, IL-6, IL-12, IL-17 A/F, IL-22, type I IFN, and GM-CSF) validate the essential role of these cytokines in human host defence, revealing their importance in coordinating antifungal defences [218].

### **Anti-GM-CSF Autoantibodies in Invasive Aspergillosis**

Although autoimmune pulmonary alveolar proteinosis (PAP) has long been the prototypical disease of anti-GM-CSF autoantibodies, this acquired phenocopy is now recognised in several settings of severe fungal disease. A recent study described a putatively immunocompetent adult with isavuconazole-refractory sino-orbital aspergillosis and high-titre neutralising anti-GM-CSF autoantibodies in the absence of PAP [219]. Mechanistically, the patient’s neutrophils had no intrinsic antifungal defect. The serum inhibited GM-CSF-mediated neutrophil priming and *Aspergillus*-induced ROS production; therefore, a cell-extrinsic, reversible defect in myeloid effector function. The case illustrates that anti-GM-CSF autoantibodies should be sought in adults with invasive mould disease without classical risk factors, even in the absence of pulmonary involvement.

### **Anti-IL-23 Autoantibodies**

Neutralising autoantibodies against IL-23 have recently emerged as drivers of severe adult-onset opportunistic infection. In a landmark three-cohort study, established an association between neutralising anti-IL-23 and severe mycobacterial, bacterial, or fungal disease [220]. In the discovery cohort, half (15 of 30) of patients with pre-existing anti-IL-12 autoantibodies and severe opportunistic infection also harboured neutralising anti-IL-23, and the potency of neutralisation correlated with infection severity. Further, thymoma patients served as a validation cohort, with strong concordance observed between anti-IL-23 and infection status. Most strikingly, in an expansion cohort consisting of patients with disseminated, cerebral, or pulmonary opportunistic infections, neutralising anti-IL-23 autoantibodies were detected in 30 of 36 patients (83%). The infection spectrum ranged from invasive mould and dimorphic fungus disease to cerebral phaeohyphomycosis due to *Cladophialophora bantiana*, and disseminated *Mycobacterium avium* complex. Anti IL-23 autoantibodies were additionally identified in 6 of 32 patients (19%) with severe intracellular infections and in 2 of 16 patients (12%) with unusual intracranial infections from the same cohort. Mechanistically, these antibodies neutralise IL-23-driven STAT3 phosphorylation in Th17 cells, thereby impairing IL-17 and IL-22 production at mucosal and pulmonary surfaces. The clinical implication is direct: any patient with unexplained invasive mould disease, disseminated dimorphic-fungus infection, or unusual intracranial fungal infection warrants anti-IL-23 screening. Anti-cytokine autoantibody panels should now include anti-IL-23 in addition to anti-IL-12, anti-IFN-γ, anti-GM-CSF, and anti-IL-17 A and IL-17 F (Table 2).

**Table 2:** Genetic defects and phenocopies associated with susceptibility to fungal infections. A consolidated overview of gene defects, mutation types, mechanisms disrupted, the resulting disease, and characteristic fungal pathogens, grouped by immune pathway

Germline defects affecting antifungal immunity, associated mechanisms and fungal pathogens				
Pathway / Gene	Defect type	Mechanism Disrupted	Disease / Phenotype	Fungal Pathogens
*JAGN1*	Loss-of-function	NETs released but non-functional (reduced MPO content); impaired candidacidal activity	Severe congenital neutropenia	*Candida albicans*
*ITGB2* (CD18)	Loss-of-function	Compromised neutrophil migration and adhesion	Leukocyte Adhesion Deficiency type I (LAD-I)	*Aspergillus*, *Candida*
NADPH oxidase subunits (*CYBB*, *CYBA*, *NCF1*, *NCF2*, *NCF4*)	Loss-of-function	Impaired ROS production; defective apoptosis/efferocytosis; LTB₄-driven hyper-swarming	Chronic granulomatous disease (CGD)	*Aspergillus* (especially *A. nidulans*), *Rhizopus*, *Trichosporon*
CARD9 (p.Q295X, homozygous)	Nonsense	Loss of CARD9; impaired CLR/NF-κB signalling	CMC; CNS candidiasis	*Candida* spp. (mucocutaneous and CNS)
CARD9 (p.G72S; p.R373P)	Missense	Impaired CLR/NF-κB signalling	CMC; *Candida* meningitis	*Candida* (CNS)
CARD9 (p.Q289X; p.R101C, homozygous)	Nonsense/missense	Loss of CARD9 protein; defective IL-6 and IL-17 A production; impaired Th17 differentiation	Severe deep dermatophytosis	Dermatophytes
CARD9 (p.S23X; p.D274fsX60; p.L64fsX59)	Nonsense / frameshift	Loss of CARD9; impaired NF-κB, IL-6/IL-1β and Th17/Th22 responses; phagocytosis and ROS preserved	Subcutaneous phaeohyphomycosis	*Phialophora verrucosa* and other dematiaceous fungi
CARD9 (other loss-of-function)	Various LOF	Reduced pro-inflammatory cytokines; aberrant CD4 T-cell responses; impaired phagocyte recruitment	Extrapulmonary aspergillosis; gut dysbiosis/IBD-like disease	*Aspergillus* (extrapulmonary), *Malassezia*
*CLEC7A* (Dectin-1; Y238X, c.714T > G)	Stop-gain	Impaired surface trafficking and β-glucan binding	Mucocutaneous candidiasis, increased aspergillosis risk in HSCT	*Candida*, *Aspergillus*
*IKBA* (NFKBIA)	Hypermorphic (gain-of-function)	Stabilises IκBα; prevents phosphorylation-induced degradation	EDA-ID	*Candida*, *Pneumocystis*
*IKBKG* (NEMO)	Hypomorphic (loss-of-function)	Impaired IKK complex assembly; reduced NF-κB activation	EDA-ID, MSMD-like phenotype	*Candida*, *Pneumocystis*
*RELB*	Biallelic loss-of-function	Disrupted non-canonical NF-κB; failure of thymic AIRE-driven negative selection (secondary anti-type-I-IFN autoantibodies)	Combined immunodeficiency	*Candida*, opportunistic fungi
*STK4* (MST1)	Autosomal-recessive nonsense	Reduced T-cell numbers; increased FAS-mediated apoptosis; impaired leukocyte migration; impaired FOXO/DAF-16–driven epidermal ROS	CVID-like combined immunodeficiency	*Candida* (CMC)
*IL17RA* (c.850 C > T; p.Q284X)	Homozygous nonsense	Loss of IL-17RA expression; unresponsive to IL-17 A & IL-17 F	AR CMC with staphylococcal skin disease	*Candida*
*IL17F* (c.284 C > T; p.S65L)	Heterozygous missense (dominant-negative)	Reduced cytokine production in leukocytes, fibroblasts, keratinocytes	AD CMC (incomplete penetrance)	*Candida*
*IL17RC*	Loss-of-function / duplication variants	Impaired IL-17 A/F signalling only; antifungal - not antistaphylococcal – defect	AR CMC without staphylococcal disease	*Candida*
*ACT1* (TRAF3IP2; c.1607 C > T; p.T536I)	Homozygous missense in SEFIR domain	Impairs ACT1 recruitment to IL-17RA/RB/RC; abolishes IL-17 A/F and IL-17E signalling	AR CMC	*Candida*
*IL23R* (p.R381Q, homozygous)	Hypomorphic missense	Severely impairs IL-23–driven STAT3 phosphorylation and Th17 cytokine output	CMC	*Candida*
*IFNGR1* or *IFNGR2*	Homozygous null	Complete loss of IFN-γ responsiveness	MSMD	Dimorphic fungi (*Coccidioides*, *Histoplasma*)
*IL12RB1*	Loss-of-function	Impaired IL-12 and IL-23 signalling; defective Th1 and Th17 immunity	MSMD + CMC	*Candida* (CMC)
*IL12RB2*	Missense	Impaired IL-12 signalling	MSMD	*Coccidioides*, *Histoplasma*, *Paracoccidioides*
*IL2RG* (common γ-chain)	Loss-of-function	Absent T and NK cells; non-functional B cells	X-linked SCID	*Pneumocystis jirovecii*, *Candida*
*CD40LG* / *CD40*	Loss-of-function	Impaired class-switch recombination and macrophage activation	Hyper-IgM syndrome (HIGM1/HIGM3)	*Pneumocystis jirovecii*, *Cryptococcus*, *Talaromyces marneffei*
*STAT1*	Autosomal-dominant gain-of-function	Aberrant STAT1 nuclear dephosphorylation; cytosolic hyperphosphorylation; impaired Th17 differentiation	CMC, autoimmunity, broad infection susceptibility	*Candida* (CMC), *Fusarium*, *Coccidioides*, *Histoplasma*
*RORC*	Biallelic loss-of-function	Impaired RORγt-driven Th17 differentiation	Mild CMC; MSMD	*Candida*
*STAT3*	Heterozygous missense / in-frame deletions (dominant-negative)	Impaired Th17 differentiation; loss of IL-17/IL-22 production	AD Hyper-IgE syndrome (Job syndrome)	*Candida* (CMC), dermatophytes, *Aspergillus*, *Histoplasma*, *Pneumocystis*
*GATA2*	Deletions, missense, frameshift	Defective GATA2; loss of monocytes, DCs, NK cells	MonoMAC syndrome / Emberger syndrome	*Histoplasma capsulatum*, *Aspergillus*, *Cryptococcus neoformans*
*IRF8* (AR, K108E)	Autosomal-recessive	Loss of circulating monocytes and dendritic cells	MSMD + combined immunodeficiency	*Candida* (CMC)
*IRF8* (AD, T80A)	Autosomal-dominant	Selective depletion of conventional dendritic cells (cDC1)	Disseminated BCG; CMC	*Candida*
*DOCK8*	Loss-of-function	Defective TH17 maintenance; impaired cytotoxic antiviral responses and IL-17–mediated antifungal defence	AR Hyper-IgE syndrome	*Candida* and other fungi; severe HPV, HSV, EBV, molluscum co-infection
*Phenocopies of IEIs predisposing to fungal infections*				
Anti–IFN-γ autoantibodies	Acquired (adult-onset)	Neutralisation of IFN-γ signalling	Phenocopy of IL-12/IFN-γ axis defect	*Talaromyces marneffei*, *Histoplasma*
Anti–GM-CSF autoantibodies	Acquired (adult-onset)	Neutralisation of GM-CSF–dependent myeloid priming	Phenocopy; PAP and invasive mould risk	*Cryptococcus gattii*, *Aspergillus*
Anti–IL-17 A/F & anti–IL-22 autoantibodies	Acquired (thymoma; APS-1)	Neutralisation of Th17 effector cytokines	Phenocopy of IL-17 pathway defects	*Candida* (CMC)
Anti–IL-23 autoantibodies	Acquired (thymoma)	Neutralisation of IL-23–driven STAT3 phosphorylation in Th17 cells	Phenocopy; severe invasive opportunistic infection	*Aspergillus*, *Histoplasma*, *Cryptococcus*, *Cladophialophora bantiana*

## Targeting the Immune Defect: Translating Insights from IEIs into Precision Diagnosis and Management of Fungal Infections

IEIs that predispose to fungal infections are powerful tools for deciphering the molecular architecture of human antifungal immunity. The rapid pace of mechanistic discovery has, in turn, translated into actionable diagnostic and therapeutic strategies. In this section, we summarise (i) when to suspect an IEI in a patient with fungal disease, (ii) a pragmatic tiered diagnostic algorithm, and (iii) IEI-specific therapy, including targeted immunomodulation and indications for haematopoietic stem cell transplantation (HSCT).

## Clinical Red Flags for When to Suspect an IEI

A high index of suspicion for an underlying inherited or acquired IEI should be maintained in several clinical settings. The first is chronic or recurrent mucocutaneous candidiasis at any age, particularly when refractory to standard antifungal therapy. Also, CMC without an obvious predisposing factor, such as recent broad-spectrum antibiotics, inhaled corticosteroids, denture use or poorly controlled diabetes. The second is fungal meningitis, fungal CNS disease, or extrapulmonary aspergillosis in the absence of classical immunosuppression. A third red flag is deep dermatophytosis, subcutaneous phaeohyphomycosis, or invasive disease caused by a fungus otherwise of low virulence. Disseminated dimorphic mycoses, such as talaromycosis, histoplasmosis, coccidioidomycosis, or paracoccidioidomycosis, should initiate prompt investigation as well. Disseminated cryptococcosis in an HIV-negative individual without solid-organ or HSCT should similarly warrant investigation. *Pneumocystis jirovecii* pneumonia in the absence of HIV infection or iatrogenic immunosuppression is similarly suspicious. In neonates, *Pneumocystis jirovecii* pneumonia is considered virtually pathognomonic of SCID. Fungal disease accompanied by recurrent skin, sinopulmonary, or invasive bacterial infection, severe atopy, autoimmunity, ectodermal dysplasia, skeletal abnormalities, or unusual viral disease (HPV, HSV, EBV-driven lymphoproliferation, molluscum contagiosum) is another important pattern. Finally, a family history of consanguinity, early death from infection, or a known IEI in a relative warrant the same diagnostic approach.

Anti-cytokine autoantibodies should be considered in any late-onset, otherwise unexplained fungal presentation, as detailed in the previous section. Anti-IFN-γ autoantibodies are characteristically associated with disseminated NTM and *Talaromyces marneffei* infection in HIV-negative adults of Southeast Asian ancestry. Anti-GM-CSF autoantibodies were originally defined as the cause of autoimmune PAP but are now also recognised in adults with HIV-negative cryptococcal CNS disease and invasive aspergillosis without classical risk factors. Wishart et al. described high-titre neutralising anti-GM-CSF autoantibodies, in the absence of PAP, in a putatively immunocompetent adult with isavuconazole-refractory sino-orbital aspergillosis [219]. Authors demonstrated that this acquired phenocopy can present with invasive mould disease in the absence of its classical respiratory manifestation. Anti-IL-17 A/F and anti-IL-22 autoantibodies should be sought in CMC associated with thymoma or AIRE-deficient APS-1. Also, anti-IL-23 autoantibodies should be tested in patients with thymoma-associated severe opportunistic infection [220].

## A Tiered Diagnostic Algorithm

After excluding acquired immunodeficiency (HIV, iatrogenic immunosuppression, malignancy, malnutrition), a stepwise approach is recommended.

### **Tier 1: Basic Immunological Screen**

Complete blood count with differential and morphology; flow cytometric enumeration of lymphocyte subsets (CD3, CD4, CD8, CD19, CD16/56) including naive/memory T-cell subsets.

### **Tier 2: Advanced Functional Testing**

Dihydrorhodamine (DHR) flow cytometric assay for NADPH oxidase function; STAT1 phosphoflow (IFN-γ, IFN-α, IL-27 stimulation) for STAT1 GOF; STAT3 phosphoflow (IL-6, IL-21 stimulation) for STAT3-LOF/HIES; Th17 enumeration (CD3⁺ CD4⁺ CCR6⁺ IL-17 A⁺) by combined surface and intracellular cytokine staining after PMA/ionomycin stimulation; IL-12/IFN-γ axis functional assays (whole-blood IL-12 production after BCG + IFN-γ stimulation; IFN-γ production after BCG + IL-12 stimulation; IFN-γ induced STAT1 phosphorylation; and surface CD119/IL-12Rβ1 expression); surface CD18/CD11b for LAD-I; specific anti-cytokine autoantibody assays (anti-IFN-γ, anti-GM-CSF, anti-IL-12, anti-IL-17 A/F, anti-IL-22, anti-IL-23, anti-type I IFN, anti-IL-6); and immunoglobulin/B-cell/T-cell subset deep phenotyping where indicated.

### **Tier 3: Genetic Confirmation**

For patients with a strongly suggestive clinical phenotype with or without functional deficiencies, targeted gene mutation analysis or gene-panel sequencing (now widely available with panels of 400–500 IEI genes) is appropriate. For phenotypically ambiguous cases or where panel testing is negative but clinical suspicion remains high, whole-exome or whole-genome sequencing should be pursued. Trio-WES (proband plus both parents) is preferred where available, as de novo and recessive variants can be identified rapidly. Variant interpretation should follow ACMG/AMP guidelines, with functional validation if needed. The 2024 IUIS classifications catalogue 559 IEIs, including 67 new monogenic defects and 2 phenocopies, and should be used as the reference framework for variant curation [19, 221].

## IEI-management

In selected IEIs, targeted immunomodulatory therapies have shifted management from infection rescue to addressing the underlying immune defect.

### ***STAT1*Gain-of-function**

Targeted JAK inhibition has transformed management. Oral ruxolitinib (JAK1/JAK2 inhibitor) competitively inhibits the ATP-binding site of JAK1/2, dampening STAT1 hyperphosphorylation and improving the clinical phenotype, often with partial restoration of Th17 numbers. Clinical remission has been achieved in children with severe CMC complicated by bacterial and *Aspergillus* co-infection, and in adults with refractory disease [222, 223].Baricitinib (JAK1/2) has produced similar symptomatic improvement in CMC. Critical clinical caveats: (i) adjunctive antifungal prophylaxis must continue throughout therapy, breakthrough fungal infections, including pulmonary coccidioidomycosis, have been reported during JAK-inhibitor therapy; (ii) reactivation of herpesviruses (VZV, HSV, CMV) is well described, and acyclovir prophylaxis should be considered; (iii) ruxolitinib is a strong CYP3A4 substrate and dose reduction is required when co-administered with triazole antifungals (voriconazole, posaconazole, isavuconazole); (iv) live vaccines are contraindicated during therapy; and (v) cytopenias are common, dose-dependent, and may require dose reduction. An observation of complete remission of oral candidiasis during anti-PD-1 therapy for head-and-neck cancer in a STAT1 GOF hints at the therapeutic potential of immune checkpoint modulation [224]. A combined EBMT-IEWP-PIDTC (European Society for Blood and Marrow Transplantation Inborn Errors Working Party (EBMT-IEWP) and Primary Immune Deficiency Treatment Consortium (PIDTC)) analysis examined 36 patients undergoing 40 HSCT procedures between 2010 and 2023 [225]. Overall and event-free survival were markedly improved compared with earlier reports. JAK inhibitor bridging therapy was associated with improved event-free survival, supporting pre-transplant immune control [225, 226]. A Hematopoietic Cell Transplantation Comorbidity Index (HCT-CI) score ≥ 1 was associated with worse outcomes [225].

### *STAT3* Loss-of-function

Management focuses on aggressive treatment of skin and sinopulmonary infections and on eczema control [227]. Fluconazole prophylaxis for CMC; mould-active triazoles (e.g. itraconazole, posaconazole) are recommended in patients with structural lung disease (pneumatoceles, bronchiectasis) and patients at risk of invasive Aspergillus disease. Dupilumab (anti–IL-4Rα) is highly effective for the severe atopic dermatitis of STAT3-HIES and can be safely co-administered with antifungal prophylaxis [228]. Demodicosis has been described and warrants periodic dermatological review. Immunoglobulin replacement does not consistently benefit patients and should be reserved for individuals with documented impaired specific antibody responses or recurrent invasive bacterial infections. A worldwide study of 41 patients who underwent transplantation reported a 5-year overall survival of 93% and an event-free survival of 90% [229]. Remarkably, 87% of patients had no or reduced bacterial or fungal respiratory infections post-HSCT, despite 32% having had pre-HSCT pulmonary fungal disease. Extra-immune manifestations are not corrected by HSCT, underscoring the need for pre-HSCT control of bronchiectasis, pneumatocele-related infection, and atopic disease.

### *CARD9* Deficiency

Long-term, prophylactic antifungal therapy with activity against both yeasts and moulds is the cornerstone of management. Granulocyte-macrophage colony-stimulating factor (sargramostim) has emerged as a promising adjunctive immunotherapy in relapsing CNS candidiasis. This acts by enhancing myeloid effector function and partially bypassing the CARD9 signalling defect. Allogeneic HSCT may be considered in patients with extensive, refractory or recurrent fungal infection [230, 231].

### *DOCK8* Deficiency

Allogeneic HSCT is the only curative option and should be pursued early, before malignancy or end-organ damage. Pre-HSCT optimisation with dupilumab and rituximab (in EBV-positive patients) has emerged as a useful bridging strategy, with reports describing clinical stabilisation and successful transplant after individualised pre-treatment. Haploidentical HSCT has been successfully performed where matched donors are unavailable. Older patients with established malignancy or refractory infection remain a transplant-management challenge, underscoring the importance of early referral [232, 233].

### **CGD**

Without a curative allogeneic HSCT, lifelong antifungal (itraconazole or posaconazole) and antibacterial (trimethoprim-sulfamethoxazole) prophylaxis is standard of care [234–236]. Subcutaneous IFN-γ (50 µg/m² three times weekly) reduces the overall infection rate. Hyperinflammatory and granulomatous complications may require corticosteroids or other immunomodulation, with the well-recognised paradox that the same patient may require both antifungal escalation and immunosuppression. Allogeneic HSCT is curative for CGD and should be discussed early. Outcomes have improved markedly across HLA-matched sibling, matched-unrelated, and haploidentical donor sources. Gene therapy is advancing rapidly: an in vivo helper-dependent adenoviral vector targeting CYBB (EN-374, Ensoma; NCT06876363) entered Phase 1/2 trials in 2025 for X-linked CGD, with the first participant dosed in December 2025; and prime editing (PM359, Prime Medicine), correcting the NCF1 delGT mutation responsible for most p47phox CGD [237].

### GATA2 Deficiency

Allogeneic HSCT is the only curative option and should be performed before the development of myelodysplasia, advanced fungal or mycobacterial disease, or pulmonary alveolar proteinosis [238].The timing must be balanced between early intervention and adequate infection control. Patients are also at heightened risk of disseminated NTM infection (notably Mycobacterium avium complex) and severe HPV-driven disease, both of which should be screened for and treated before transplant. Antifungal prophylaxis (typically itraconazole or posaconazole) is recommended once the diagnosis is established, given the high incidence of disseminated histoplasmosis, invasive aspergillosis, blastomycosis, and cryptococcosis [239, 240].

### IL-12/IFN-γ Axis Defects

Few case series have used recombinant IFN-γ (typically 50 µg/m² three times weekly) to augment macrophage activation and fungal clearance in selected patients with defects in IFN-γ production. This is mainly useful for patients with IL-12p40 and IL-12Rβ1 deficiencies, in which the receptor remains intact and exogenous cytokine signalling is normal. It is ineffective and should not be used in complete IFN-γR1 or IFN-γR2 deficiency, in which the receptor is absent or nonfunctional, preventing exogenous IFN-γ from transducing a signal. For receptor defects, allogeneic HSCT remains the only curative option [241, 242]. Where IFN-γ is indicated, close monitoring for treatment-related inflammatory complications is required.

### Anti-cytokine Autoantibody Disease (Acquired Phenocopies)

Management combines aggressive antimicrobial therapy with strategies to reduce the offending autoantibody. Many patients fail to respond durably or develop toxicity from prolonged antimicrobials alone. Rituximab (anti-CD20) has been used successfully in patients with high-titre neutralising anti-GM-CSF, anti-IFN-γ, or anti-IL-17 autoantibodies refractory to conventional treatment (NCT01842386). Cyclophosphamide may be considered for plasmablast-driven refractory disease. B-cell depletion carries risks of hypogammaglobulinaemia and secondary infection and should be undertaken in specialist centres. Plasmapheresis is occasionally used in fulminant presentations. Recombinant GM-CSF supplementation has been described in anti-GM-CSF disease.

### Cautions with Novel Biologics and Small Molecules

The expanding landscape of biologic therapies presents both opportunities and risks. Biologics targeting the IL-17/IL-23 axis predictably increase the risk of CMC, dermatophytosis, and candidiasis, mirroring the inborn deficiencies in the IL-17/IL-23 pathway. The relevant drugs are anti-IL-17 A (secukinumab, ixekizumab), anti-IL-17 A/F (bimekizumab), anti-IL-17RA (brodalumab), anti-IL-23p19 (risankizumab, guselkumab, tildrakizumab), and the anti-p40 agent ustekinumab, which blocks the shared IL-12/IL-23 subunit. These should be used cautiously in any patient with a history of CMC or established IEI affecting this pathway. JAK inhibitors carry recognised risks of herpes zoster reactivation and opportunistic infections. Oral pan-JAK inhibitors, notably tofacitinib at rheumatology doses, additionally carry a class warning for major adverse cardiovascular events and venous thromboembolism following the ORAL Surveillance trial [243]. Antifungal prophylaxis is appropriate during JAK inhibitor therapy in patients with IEIs. CYP3A4 interactions between triazoles and JAK inhibitors, calcineurin inhibitors, and many other agents must always be considered, and formal dose adjustments must be made.

### **Co-infections in IEIs as a Paradigm to Study Immune Cross-talk**

IEIs are natural experiments in which a single gene defect reveals the hierarchy and plasticity of host defence against diverse pathogens. Several IEIs are characterised by an obligate triad of fungal, bacterial and viral or mycobacterial co-infection that illuminates this cross-talk. These expose a recurring principle: the cytokine circuits that coordinate antifungal immunity are the same ones that govern antibacterial, antiviral, and antimycobacterial defence. Thus, a monogenic defect rarely produces a single-pathogen phenotype.

CGD harbours a paradigmatic example of fungal and bacterial pathogen coexistence. Patients may present with invasive aspergillosis (notably *Aspergillus nidulans*, almost CGD-pathognomonic), a catalase-positive bacterial disease (*Staphylococcus aureus*, *Burkholderia cepacia*, *Serratia marcescens*, *Nocardia* species), and an increasingly recognised burden of viral co-infections [26, 31]. Aspergillus-associated invasive disease remains a key determinant of mortality. *Staphylococcus aureus* causes deep-seated infections, including liver abscesses that are often refractory to drainage and respond best to combined steroid-antibiotic therapy. *Burkholderia* species cause pneumonia that may progress to bacteraemia, sepsis or haemophagocytic lymphohistiocytosis (HLH), requiring accurate species-level diagnosis and frequently combined medical-surgical management [26]. This multi-pathogen susceptibility is further amplified by the paradoxical hyperinflammatory state characteristic of CGD. In turn, this state leads to sustained innate immune activation, cytokine imbalance, and granuloma formation [28, 31, 244]. Emerging non-*Aspergillus* moulds in CGD include *Trichosporon*, *Geosmithia argillacea* and *Rasamsonia* species.

*STAT1 GOF disease* presents the broadest co-infection profile of any IEI. Beyond CMC, *STAT1 GOF* patients exhibit susceptibility to invasive fungal, bacterial, viral and mycobacterial infections, with viral infections occurring in approximately 38% and invasive fungal disease in about 10% of cases, underscoring the unusually broad infectious phenotype associated with this disorder [245]. Consequently, STAT1 GOF is now regarded as a syndromic form of chronic mucocutaneous candidiasis (SCMC). Infections such as CMC, recurrent staphylococcal disease, mycobacterial infections, *Aspergillus* infection, and a strikingly high rate of clinically significant herpesvirus reactivation (VZV meningitis and retinitis, ulcerative CMV oesophagitis, HSV encephalitis) may be present. Toubiana et al. reported this fungal-bacterial-viral co-infection pattern in detail in an international cohort. Mechanistically, STAT1 GOF mutations impair nuclear dephosphorylation of STAT1, producing sustained phosphorylation and exaggerated responses to type I and II interferons and IL-27 [246]. This hyperactive STAT1 signalling inhibits Th17 differentiation by antagonising the STAT3/RORγt axis. This impairs IL-17-mediated mucosal immunity, which underlies the CMC phenotype and contributes to a broader infectious spectrum [177, 187]. Approximately one-third of STAT1 GOF patients develop clinically significant herpesvirus complications alongside CMC, offering a clear window for fungal-viral-immune cross-talk [193]. DOCK8 deficiency pairs mucocutaneous *Candida* with severe HSV, HPV, EBV-driven lymphoproliferation, and molluscum, reflecting a shared cytoskeletal requirement for both antiviral cytotoxicity and antifungal Th17 responses.

*IL-12/IFN-γ* axis defects lead to the classical fungal-mycobacterial pairing of dimorphic fungal disease (talaromycosis, histoplasmosis, coccidioidomycosis, paracoccidioidomycosis) with non-tuberculous mycobacterial infection. Common variable immunodeficiency (CVID) drives fungal-bacterial co-infection, including *Pneumocystis jirovecii* pneumonia and recurrent encapsulated bacterial sinopulmonary infections. Acquired phenocopies reproduce these combinations almost exactly: in Southeast-Asian adults, anti-IFN-γ autoantibodies produce a distinctive triad of disseminated non-tuberculous mycobacterial disease, *Talaromyces marneffei* infection, and disseminated salmonellosis that recapitulates inborn IL-12/IFN-γ axis defects. Indeed, recent international series establish that *T. marneffei* infection in an HIV-negative patient should prompt evaluation for an inborn or acquired defect of the IL-12/IFN-γ axis or STAT3-dependent Th17 immunity [152, 247]. Together, these examples make a broader point: monogenic IEIs (and their acquired phenocopies) reveal that susceptibility to a given pathogen is not a binary loss-of-function but a dynamic state shaped by cytokine cross-talk, cell-type-specific signalling thresholds, and the timing of prior infections. The systematic study of IEI co-infections may therefore inform the rational selection of biologic and small-molecule therapies, as well as the design of host-directed antifungal strategies.

**Box 5** Dysregulated immune response as a driver of pathology.

**Table:** 

Importantly, not all immune responses are protective; dysregulated immunity can itself drive fungal pathology, and this principle recurs across organisms and tissues. In HIV-associated cryptococcal meningitis, an exaggerated neutrophilic response correlates with increased mortality. It was seen that a baseline neutrophil count above 3,500 cells/µL confers a higher 30-day and 12-month mortality in a cohort of 801 patients [248]. The same duality is seen beyond the CNS. Neutrophil extracellular traps formed during invasive pulmonary aspergillosis can aggravate acute lung injury while paradoxically limiting fungal clearance. Also exemplified in immune reconstitution inflammatory syndrome (IRIS), which is described with Cryptococcus, Aspergillus, Histoplasma, Candida, and Pneumocystis. In IRIS, clinical worsening is observed even as the fungal burden declines. Tissue resident macrophages illustrate the same point: rather than clearing Cryptococcus neoformans, microglia can serve as an intracellular reservoir. It was observed that brain-infiltrating CD4 + T cells may amplify a dysfunctional, poorly fungicidal microglial state in a murine model of cryptococcosis [249, 250]. Thus, the balance between protective and pathological inflammation is delicate and context dependent. It reinforces the fact that immune cells indispensable in one setting may become liabilities in another.
Another example of a protective response becoming pathological is neutrophil swarming in patients with CGD. Despite the ROS deficit in the absence of NADPH oxidase, neutrophil swarming is amplified around fungal foci. Neutrophil swarming is an organised, regulated movement of neutrophils toward infection. It involves rapid aggregation at infection sites driven by leukotriene LTB₄- and Ca²⁺-dependent feed-forward loops triggered by pathogen or damage-associated molecular patterns [251]. Under normal conditions, NADPH oxidase-generated ROS depolarises the neutrophil membrane to limit Ca²⁺ influx, while GRK2 desensitises BLT1 (the LTB₄ receptor) to terminate amplification and form self-limited swarms. In CGD, loss of NADPH oxidase activity removes this regulatory checkpoint. Membrane hyperpolarisation causes Ca²⁺ overload, excess LTB₄ release, and BLT1-driven hyper-swarming, culminating in pathological hyperinflammation. Song et al. demonstrated that CGD neutrophils produce elevated levels of LTB₄ following zymosan stimulation and form larger BLT1-dependent clusters in vitro, thereby directly confirming this mechanism. In vivo, CGD mice develop extensive pulmonary neutrophilic infiltrates after fungal challenge that are attenuated by LTB₄ synthesis inhibitors or BLT1 antagonists [252]. An additional IL-1β/G-CSF circuit further amplifies tissue infiltration by mobilising immature neutrophils from bone marrow [253]. Together, these findings identify NADPH oxidase as the key regulatory checkpoint constraining neutrophil swarming and position the LTB₄-BLT1 axis as a promising host-directed therapeutic target in CGD.

## Future Directions

While this review has focused on well-characterised immune defects, IEIs provide a powerful framework not only for understanding single-pathogen susceptibility but for dissecting the shared immune circuits that underlie co-infections [254]. Historically, IEIs have been classified by the dominant pathogen phenotype, such as IL-17RA deficiency predisposing to *Candida* infections and IFN-γR deficiency predisposing to *Mycobacterium* infections. Yet these same pathways extensively intersect in regulating antifungal, antibacterial, and antiviral responses. Studying genetically defined IEIs through the lens of their co-infections is likely to reveal how host defence is prioritised and reconfigured when multiple effector arms are simultaneously engaged.

Two areas are particularly underserved. First, the burden of inborn and acquired immune defects underlying endemic mycoses in low and middle-income countries (LMIC) remains largely uncharted. This is exemplified by anti-IFN-γ autoantibody disease, which was recognised in 2004 and is predominantly seen in Asian-born individuals, highlighting how a major susceptibility can persist for two decades without large-scale characterisation. Second, the boundary between monogenic immunodeficiency and acquired phenocopy is proving more porous than previously assumed, as disorders such as RELB deficiency now demonstrate.

Future studies should prioritise: (i) prospective IEI-fungal registries capturing genotype, pathogen, treatment, and outcome; (ii) standardised functional immune phenotyping paired with sequencing; (iii) systematic screening for anti-cytokine autoantibodies in adult-onset or unexplained invasive fungal disease; and (iv) LMIC and endemic-mycosis cohorts in which careful immunological work-up of talaromycosis, histoplasmosis, coccidioidomycosis, and paracoccidioidomycosis may uncover underdiagnosed IEIs. As genetic diagnosis becomes faster and cheaper and pathway-targeted therapies mature, the systematic study of these vulnerabilities will increasingly translate into precision immunotherapy across diverse infectious contexts.

## Data Availability

No datasets were generated or analysed during the current study.
